# Wearable Pulse Wave Monitoring System Based on MEMS Sensors

**DOI:** 10.3390/mi9020090

**Published:** 2018-02-23

**Authors:** Yu Sun, Ying Dong, Ruyi Gao, Yao Chu, Min Zhang, Xiang Qian, Xiaohao Wang

**Affiliations:** 1 Graduate School at Shenzhen, Tsinghua University, University Town of Shenzhen, Shenzhen 518055, China; sy15@mails.tsinghua.edu.cn (Y.S.); gry17@mails.tsinghua.edu.cn (R.G.); zhang.min@sz.tsinghua.edu.cn (M.Z.); qian.xiang@sz.tsinghua.edu.cn (X.Q.); wang.xiaohao@sz.tsinghua.edu.cn (X.W.); 2 Tsinghua-Berkeley Shenzhen Institute, University Town of Shenzhen, Shenzhen 518055, China; chuy15@mails.tsinghua.edu.cn

**Keywords:** MEMS sensor, wearable device, pulse wave, signal processing

## Abstract

Pulse wave monitoring is critical for the evaluation of human health. In this paper, a wearable multi-sensor pulse wave monitoring system is proposed and demonstrated. The monitoring system consists of a measuring unit and an analog circuit processing unit. The main part of the measuring unit is a flexible printed circuit board (PCB) with a thickness of 0.15 mm, which includes three micro-electromechanical system (MEMS) pressure sensors softly packaged by polydimethylsiloxane (PDMS), a blood oxygen detector and a MEMS three-axis accelerometer. The MEMS pressure sensors，the blood oxygen detector and the accelerometer are fixed on the expected locations of the flexible PCB. The analog circuit processing unit includes a power supply module, a filter and an amplifier. The pulse waves of two volunteers are detected by the monitoring system in this study. The output signals of the analog circuit processing module are processed and analyzed. In the preliminary test, the time delay of the three pressure pulse waves has been detected and the calculated pulse wave velocities (PWVs) are 12.50 and 11.36 m/s, respectively. The *K* value, related to the area of the pulse wave, can be obtained. Both the PWV and *K* value meet the health parameter standards.

## 1. Introduction

Pulse diagnosis is one of the important diagnostic techniques in traditional Chinese medicine. The theory of pulse diagnosis has been applied and studied by many Chinese medicine practitioners. Many scholars have studied the pulse wave and quantitatively analyzed pulse wave information, including velocity, amplitude and so on, which is closely related to some diseases of the human body, such as diabetes, renal disease and arteriosclerosis [1,2,3,4,5]. The pulse wave contains a wealth of blood flow information. The pulse wave presents comprehensive information on the waveform, wave velocity, amplitude and cycle which, to a large extent, reflects many physiological and pathological characteristics of the human cardiovascular system. The radial artery is the best place to obtain the pressure pulse wave. With one’s fingers placed on the wrist, one can feel the pulse of the artery, which is the traditional way of Chinese medicine pulse diagnosis. The pulse wave information of the radial artery is particularly rich and the detection of the radial artery pulse wave is convenient due to it being close to the skin surface.

Although there are pulse monitoring products on the market, they are complex in structure and large in volume. The available pulse monitoring products are difficult to carry, and they also cannot be used for continuous, real-time monitoring. In recent years, more and more scholars have begun to study wearable devices for human health evaluation [6,7,8,9,10,11]. There are also some wearable devices on the market for detecting the pulse. Most of these devices are intelligent hand rings, such as Jawbone UP2, Fitbit Force, Apple Watch and so on. However, most of the wearable devices are bracelet structures, so the positioning accuracy of the device is low, and can only be used for detecting the pulse frequency, but not for detecting the entire pulse. However, overall, the detection, recording and analysis processing of the pulse wave is constantly updated and improved. With the development of MEMS sensors, the advantages of miniaturization, integration, high sensitivity, low energy consumption, excellent mechanical and electrical performance of MEMS sensors are becoming more and more prominent. It is expected that such technologies will potentially be able to revolutionize the health care industry and transform current care delivery and treatment decisions from the health care system to the individual. Yu-Pin Hsu proposed and demonstrated a prototype skin-coupled personal wearable ambulatory pulse wave velocity (PWV) monitoring system [4]. Reference [7] reported a microfluidics-based flexible interfacial capacitive sensor that can be attached on a wearer’s neck area to detect blood pressure pulses. McCombie used a method with two photoplethysmograph (PPG) sensors at a known distance [8]. David M.D. Ribeiro proposed a wearable electrocardiogram (ECG) and continuous blood pressure monitoring system for cardiovascular diseases [9]. T. Kaneko proposed a piezoresistive cantilever on liquid that can measure pulse waves at various points on the human body with high sensitivity [10]. D.B. Mccombie measured the peripheral pulse transit time with a wearable device which consists of two in-line photoplethysmograph (PPG) sensors [11]. 

**Figure 1:**
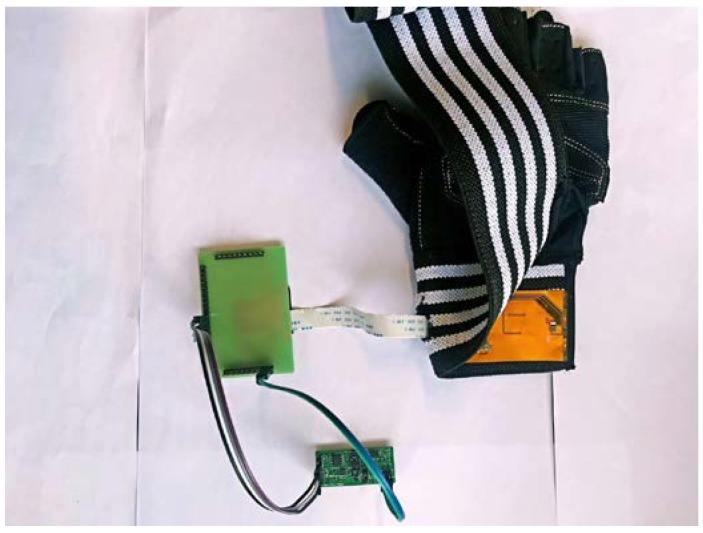
The panorama of the system.

In this work, MEMS pressure sensors are used to detect the pressure pulse wave of the radial artery. A blood oxygen detector is also used to detect the volume of the pulse wave and a MEMS accelerometer is used to monitor the movement of the wrist for compensation. The sensors are packaged with the process circuit on a flexible printed circuit board (PCB). A new glove structure is designed instead of a bracelet to increase the sensor positioning accuracy. By fixing the flexible PCB on the glove structure, a small and wearable pulse wave monitoring system has been developed. The panorama of the system is shown in Figure 1. All sensors and preprocessing circuits can be placed in the glove if part of the test is removed. The advantages of the system are its small size, wireless communication, low power consumption and real-time pulse wave detection. The resolution of the system is about 100 Pa; it is influenced by pressure sensors. The PCB includes a power module, an amplifier module, a filter module and a Bluetooth module. The power module converts 3.7 V provided by the battery to the voltages which are required by the sensors. The amplification and filter circuit pre-process the analog signals generated by the MEMS pressure sensors and the blood oxygen detector. The Bluetooth module transmits signals to the terminal. Finally, the terminal completes the pulse wave signal processing. The wave velocity and the characteristic parameters related to the pulse wave can be obtained.

## 2. Principles

### 2.1. Generation of Pulse Wave

**Figure 2:**
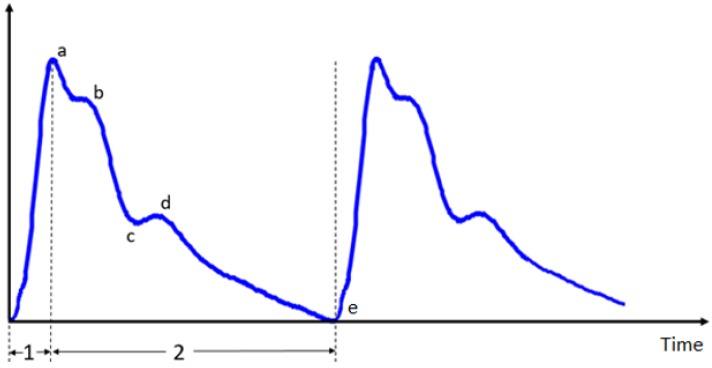
Waveform and characteristic points of the pulse wave.

The pulse wave originates from the heart; it is affected by the heart as well as various physiological factors, such as blood viscosity, vascular resistance and vascular wall elasticity of the arteries and their branches. The pulse wave contains abundant physiological and pathological information. The shape of the pulse wave is shown in Figure 2. Points a, b, c, d, e are feature points for evaluating the pulse wave, of which the height and the distance represent the health of the human body [4]. 

### 2.2. Pulse Wave Characteristic Values

Based on clinical data and mathematical models, the characteristics of the pulse wave can be obtained through extracting some physiological points in the pulse wave (as described in 2.1). Professor Luo Zhichang [5] put forward a characteristic quantity of the pulse wave, the *K* value, which is defined as:

(1) $$K=\frac{P_{m}-P_{d}}{P_{s}-P_{d}}$$

where $P_{s}$ is the systolic pressure, $P_{d}$ is the diastolic pressure, $P_{m}$ is the mean pressure. The *K* value depends on the shape of the pulse wave, and it is related to the elasticity of the vascular wall, the blood sticky and so on. The *K* value of healthy people is between 0.32 and 0.38.

**Figure 3:**
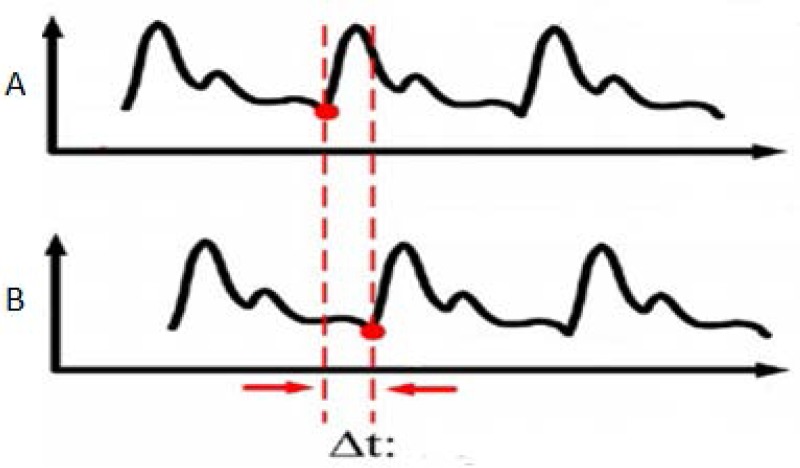
Measurement of the pulse wave delay time.

As the blood is an incompressible fluid in the arteries, the energy spread is mainly in the arterial wall rather than the blood fluid, so the pulse wave velocity (PWV) can reflect the hardness of the arterial wall [12]. In general, the greater the compliance of the arterial wall, the slower the speed of the pulse wave. The increase of vessel wall hardness can promote the pulse wave velocity. The PWV can be obtained by measuring the delay time and the distance between two measuring positions [13]. The formula is shown as:

(2) $$\mathrm{PWV}=\frac{\Delta S}{\Delta t}$$

where ΔS is a definite distance between A and B, Δt is the delay time between A and B, which is shown in Figure 3.

References [14,15] demonstrate the relationship between blood pressure and pulse wave transfer time in detail. References [2,3] use clinical data to verify the correlation between pulse wave speed and some diseases. 

## 3. Measurements

### 3.1. System Composition

The pulse wave is a weak quasi-periodic signal. The pulse wave signal has a certain complexity. The formation of the pulse wave is directly related to the contraction of the heart, and is related to other organs. During the measurement process, the pulse wave is also affected by the position of the sensing device, the physical condition of the volunteer and the movement of the wrist. To solve these problems, this paper uses a multi-sensor pulse wave monitoring system that consists of three MEMS pressure sensors, a blood oxygen detector and a three-axis MEMS accelerometer.

**Figure 4:**
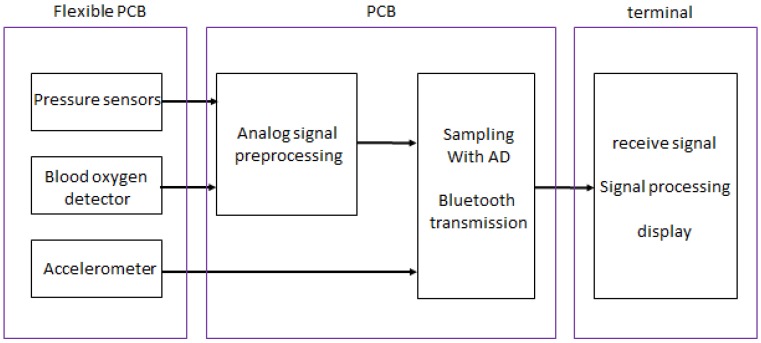
The block diagram of the monitoring system.

The three MEMS pressure sensors are placed at three measuring points of the radial artery between which the distance is certain, and then the pressure pulse waves at three points are measured simultaneously. The blood oxygen detector measures the volume pulse wave of the wrist. The three-axis accelerometer monitors the wrist's movement which is used as a motion reference for removing noise generated by movement from pulse waves. The sensors and the processing circuit are packed on a flexible PCB. The block diagram of the pulse wave monitoring system based on MEMS sensors is shown in Figure 4.

Interventionary studies involving animals or humans, and other studies that require ethical approval must list the authority that provided approval and the corresponding ethical approval code. 

### 3.2. Sensor Selection

**Figure 5:**
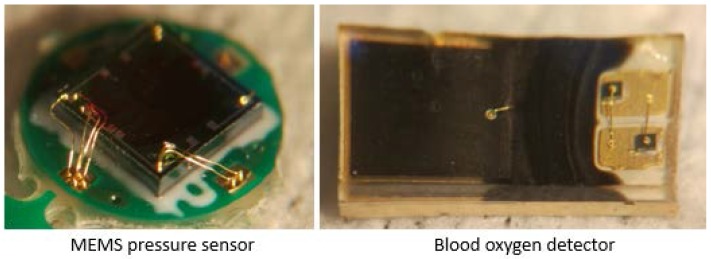
The blood oxygen detector and the packaged MEMS pressure sensor.

Sensors should be selected according to the characteristics of pulse wave signals. After comparison, the monitoring system uses three XGZU2009 piezoresistive sensor chips (CFSensor, Wuhu, China) to detect the pressure pulse wave. The size of the chip is 2 mm × 2 mm × 0.9 mm and the measurement range of the chip is 0~20 Kpa. The chip has good linearity, repeatability, stability and high sensitivity, which meet the measurement requirements commendably. The MEMS pressure sensor chips (CFSensor, Wuhu, China) are packaged with PDMS (Mixture ratio is 10:1, Dow Corning, Midland, MI, USA) to provide a comfortable skin attachment, which is highly attractive for long-term monitoring. The package can also protect the sensitive layer and lead wire. A DCM05 oximeter (Zolix, Beijing, China) is chosen to measure the blood oxygen saturation under certain conditions to detect the volume of the pulse wave. In general, the actual output consists of a small AC signal and a DC signal. The small AC signal is our target signal, caused by the pulsation of the arterial blood, while the DC signal is caused by the constant absorption of light by the blood. An ADXL335 three-axis accelerometer (Analog Devices, Norwood, MA, USA) which is small in size, has a low power consumption and larger measurement range is used to detect the movement of the wrist; it can be used for motion compensation. The sensors are shown in Figure 5.

### 3.3. Package of the MEMS Pressure Sensor

The MEMS pressure sensor mentioned above is not a fully packaged chip. Its lead wire and sensitive layer are both exposed to the exterior environment. Therefore, it cannot be directly used in contact with the skin to measure the pulse wave. In order to protect the lead wire and the sensitive layer, and make a more comfortable contact between the MEMS pressure sensor and skin, the sensors are packaged with PDMS. 

**Figure 6:**
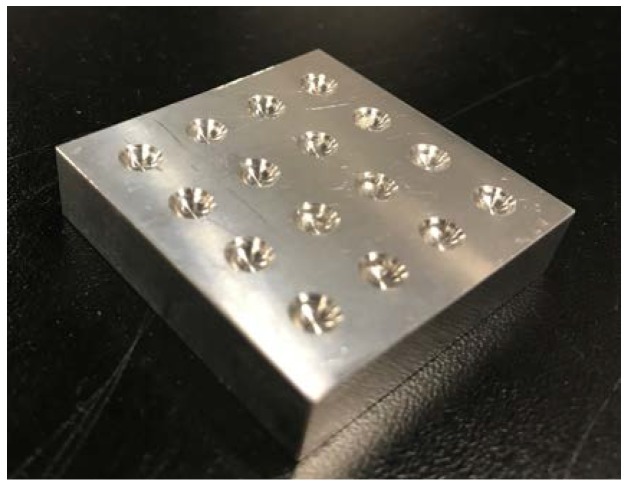
Mold for packaging MEMS pressure sensors.

PDMS and the curing agent are first mixed in a ratio of 10:1, and the mixed solution needs to be evacuated. The solution is poured into the mold as shown in Figure 6, and the MEMS pressure sensor chips are placed in grooves of the mold, whose size matches the size of the sensors. Finally, the mold is placed in the oven to be heated at a temperature of 80 °C for 2 h. Three sensors are packaged at the same time, which maintains the consistency of the sensor package and improves the accuracy of the measurement. 

### 3.4. Sensor Installation

**Figure 7:**
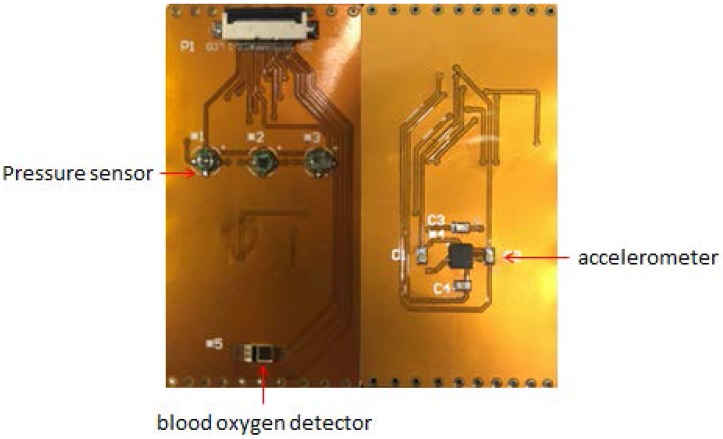
The flexible PCB.

To place the sensors in as much contact with the skin as possible, the main part of the measuring unit is designed as a flexible PCB with a thickness of 0.15 mm, which includes three MEMS pressure sensors, a blood oxygen detector and a three-axis accelerometer, as shown in Figure 7. The sensor is soldered to the PCB. The distance between the pressure sensors is constant. 

Existing wrist wearable devices can only ensure that the device is stable in the direction perpendicular to the wrist, but cannot ensure that the device is stable in the direction parallel to the wrist. The instability affects the positioning accuracy of the sensor placed in contact with the radial artery; this will affect the real-time detection. Therefore, the glove structure is designed in this work, fixing the movement in all directions, which guarantees the positioning accuracy of the sensors. The location on the glove, in relation to the radial artery, needs to be confirmed; this is the position of MEMS pressure sensors. After positioning, the flexible PCB can be effectively combined with the glove through the small holes in both sides. During the test, the sensors need to be fixed on the radial artery with manual intervention. The wrist strap of the glove can give a pretightening force between the wrist and device by winding the wrist. The glove structure is a good solution to the above problems.

## 4. Amplification and Filter Circuit

**Figure 8:**
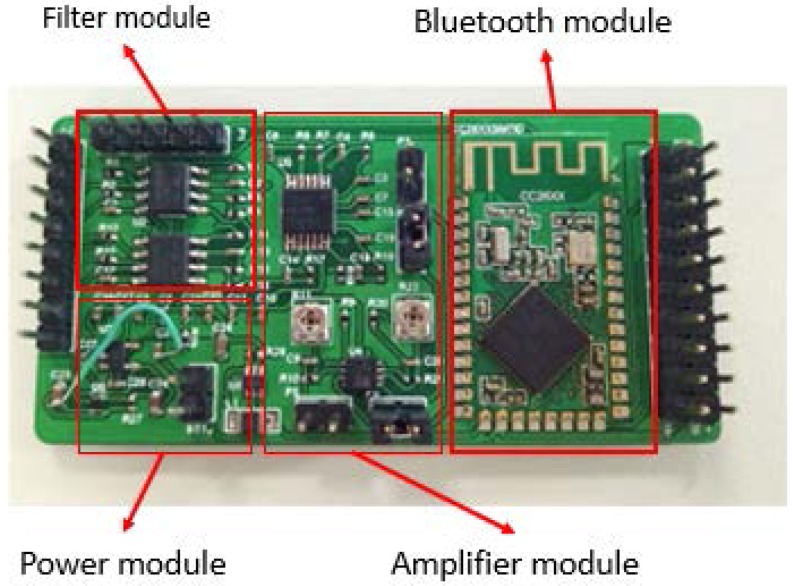
Physical picture of the hardware circuit.

Jingjing Zhang designed a hardware circuit for the Chinese pulse-wave retrieval system [16]. In order to meet the requirements of power supply, signal preprocessing, signal transmission and so on, a processing circuit was designed, which includes a power module, an amplifier module， a filter module and a Bluetooth module. The principle of the hardware circuit is relatively simple. A physical picture of the hardware circuit is shown in Figure 8.

Taking into account the operating voltage of the chip and the sensors in the circuit, the power module will convert the 3.7 V output voltage of the lithium battery into 2 V and ±5 V. The filter is designed for the fourth-order Butterworth filtering, and the cut-off frequency is designed to be 60 Hz. The amplification value of the amplifier can be freely adjusted within a certain range.

## 5. Results and Discussion

**Figure 9:**
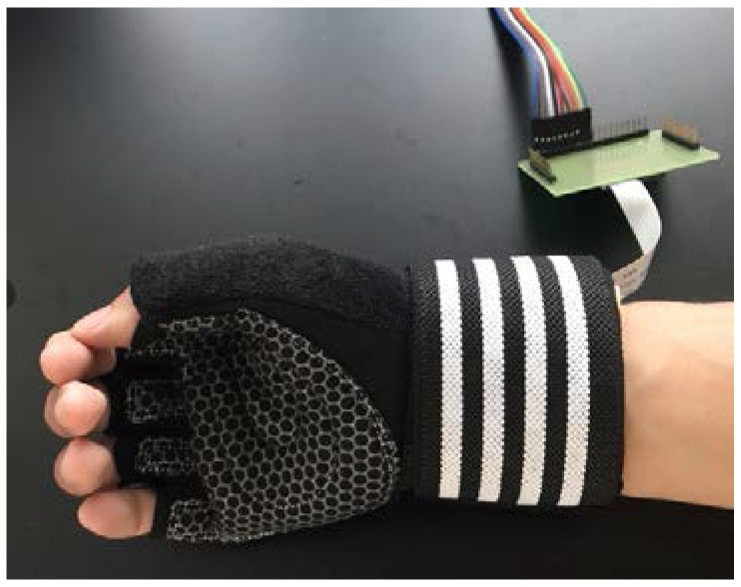
The wearable pulse wave monitoring system.

The flexible PCB is fixed on a glove to make a wearable pulse wave monitoring system. In the experiments, the glove is adjusted to suit the volunteers. The pressure pulse wave and the volume pulse wave of the radial artery and the movement state of the wrist can be detected by the wearable pulse wave monitoring system. The prototype is shown in Figure 9.

**Figure 10:**
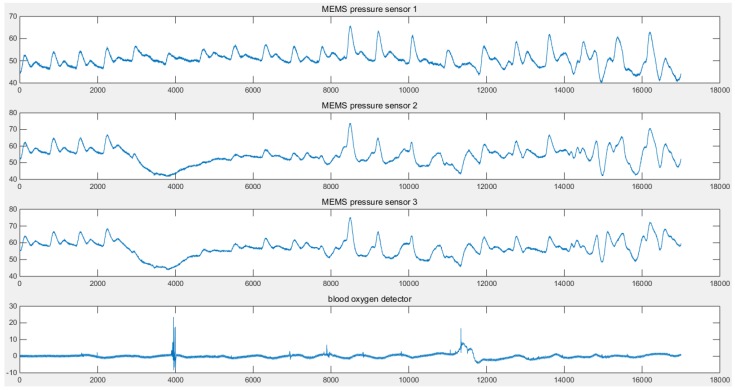
Waveforms of a multi-sensor pulse wave monitoring system.

The data is sampled with a 1 KHz signal and transmitted to the terminal. The obtained data is shown in Figure 10.

**Figure 11:**
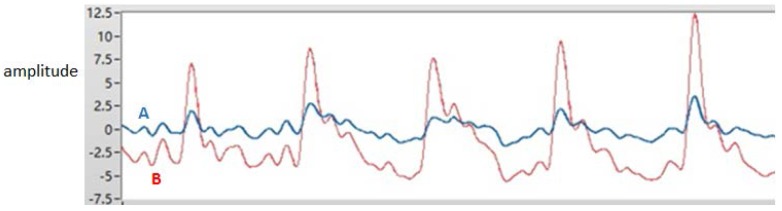
The processed pulse waves.

**Figure 12:**
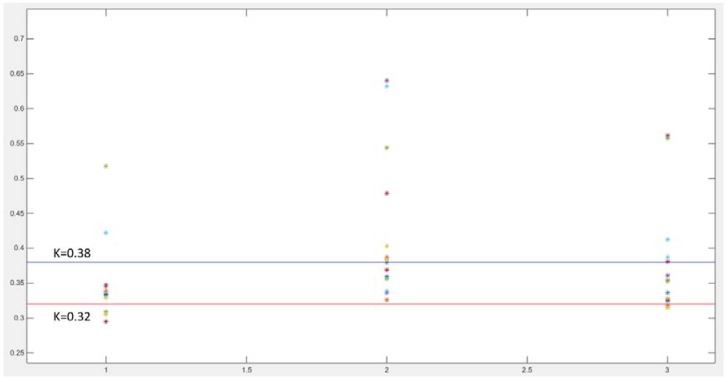
The chart of *K* values.

Sensors located at different points on the radial artery may produce signals of different quality. In this work, three MEMS pressure sensors are used to provide a selection of signals. In theory, there is a certain correlation between the volume of the pulse wave and the pressure of the pulse wave. The movement of the wrist not only affects the blood flow, but also affects the stability of the contact between the MEMS pressure sensors and the wrist. In actual measurement, the detection of the volume of the pulse wave is less affected by movement. When the data is processed, the pressure sensor signals need to be compensated by the accelerometer data. It is also necessary to use the volume pulse wave data and the pressure pulse wave data to perform the cross-correlation operation. Two signals of better quality are selected for the two measurement points. The red curve and the blue curve in Figure 10 are the pressure pulse waves of the two measurement points. The distance ΔS between the MEMS pressure sensors is constant. The signals detected by the MEMS pressure sensors—signal A and signal B respectively, as shown in Figure 11 are processed and intercepted for a certain period. There is a delay time between the two signals. The average of the delay time between two adjacent peaks is taken as the standard for the purpose of calculation. For one of the volunteers, the delay time Δt = 0.002 s. It is possible to deduce that the PWV = ΔS/Δt = 12.50 m/s. The delay time of another volunteer Δt = 0.0022 s, which is used to deduce that the PWV = 11.36 m/s. Three MEMS sensors were used to detect the health of volunteers; pressure pulse wave data was obtained which included data in the motion state. The *K* values can be obtained from the data and from Figure 12. Due to movement and other factors, there are some errors in the data. However, the measurement result is still highly accurate. The error rates are around 25%. In general, both PWVs and *K* values meet the health parameter standards.

The accuracy and consistency of the sensors are very important factors to the wearable pulse wave monitoring system. MEMS pressure sensors are packaged by PDMS with the accurate mold. Furthermore, the ratio, heating time and temperature of PDMS are strictly limited. However, this cannot completely guarantee the consistency of the sensor package, which leads to a different sensor output. By using the multi-sensor signal fusion technique and adaptive filtering, the Kalman filter and other signal processing algorithms, the waveform, wave velocity, amplitude and cycle of the pulse wave in the quiescent state can be accurately determined. However, when dealing with motion data, the resulting signal distortion is very high, and the calculated *K* value and pulse wave velocity contain considerable errors.

## 6. Conclusions

This paper presents a prototype design of a wearable multi-sensor pulse wave monitoring system. The prototype system proves that the non-invasive and real-time detection method is, to a certain extent, feasible. The wearable pulse wave monitoring system can be used for early detection and manual intervention. It is possible to improve the pulse wave detection accuracy by improving the packaging of the sensor, the stable contact of the sensor with skin, using the highly sensitive sensor and more optimal signal processing techniques. 

A sensor array will be implemented in the design of future systems to enable intelligent array scanning to select optimal sensors for real-time operation. Besides an accelerometer, gyroscopes could also be incorporated into future wearable systems to detect motion artifacts, thus enabling an effective calibration and suppression of the artifacts.
